# Supplementary material for: Structure, Physicochemical Properties and Biological Activity of Lipopolysaccharide from the Rhizospheric Bacterium Ochrobactrum quorumnocens T1Kr02, Containing d-Fucose Residues
Source: Int J Mol Sci. 2024 Feb 6;25(4):1970. doi: 10.3390/ijms25041970 (PMC10888714; doi:10.3390/ijms25041970)
Supplement: Supplementary file 1 [file ijms-25-01970-s001.zip › ijms-2852926-supplementary.pdf]

Supplemantal Materials

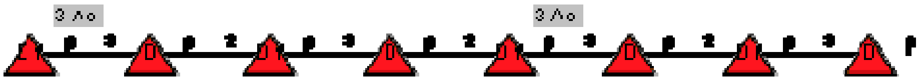

a

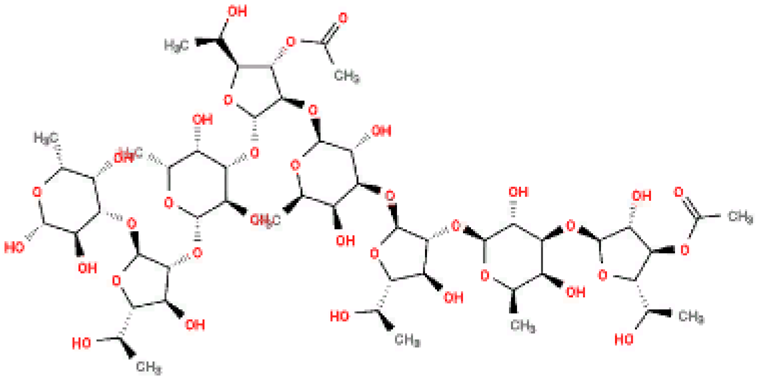

b

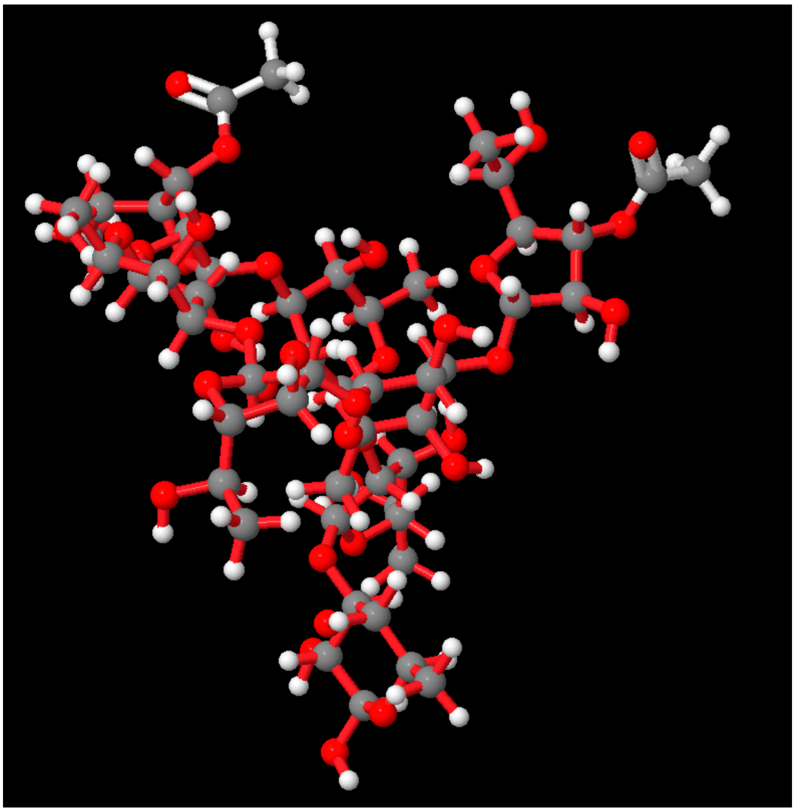

c

Figure S1. Schematic (**a**) and structure (**b**) formulas, and molecular model (**c**) of PS-1 from *O. quorumnocens* T1Kr02, containing four repeating units →2)-β-D-Fuc*f*-(1→3)-β-D-Fuc*p*-(1→.

50% of the D-Fuc*f* residues (in the second and fourth units) at position 3 are O acetylated.

Atoms (balls) are designated as follows: gray – carbon; red – oxygen; white – hydrogen. Bonds between atoms in the oligosaccharide are indicated by red sticks, and bonds in the acetyl group are indicated by white sticks.

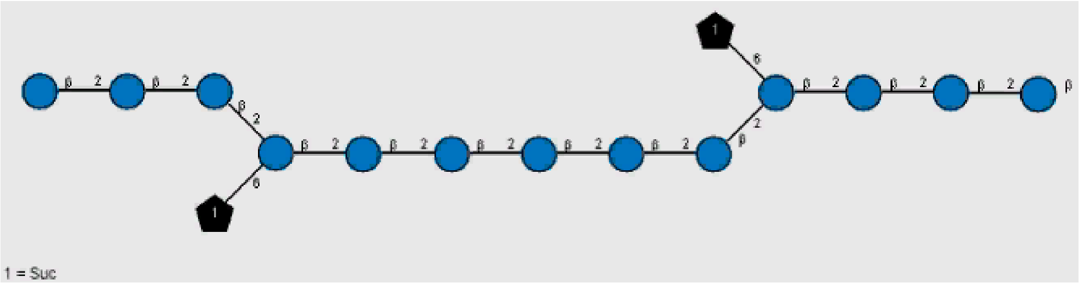

a

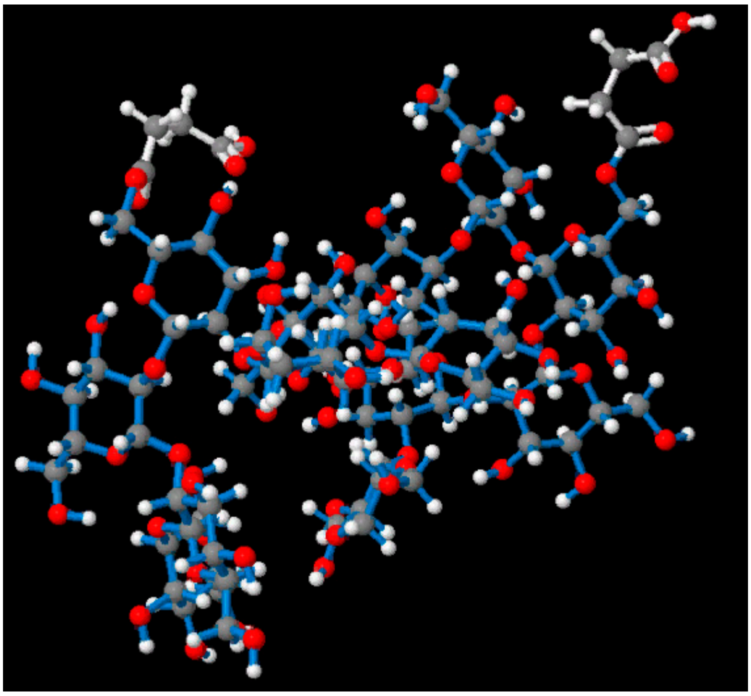

b

Figure S2. Schematic formula (**a**) and molecular model (**b**) of PS-2 from *O. quorumnocens* T1Kr02, containing thirteen repeating units →2)-β-D-Glc*p*-(1→.

Two residues of the D-Glc*p* (~15%) at position 6 are linked with a succinate residue.

Atoms (balls) are designated as follows: gray – carbon; red – oxygen; white – hydrogen. Bonds between atoms in the oligosaccharide are indicated by blue sticks, and bonds in the succinate group are indicated by white sticks.

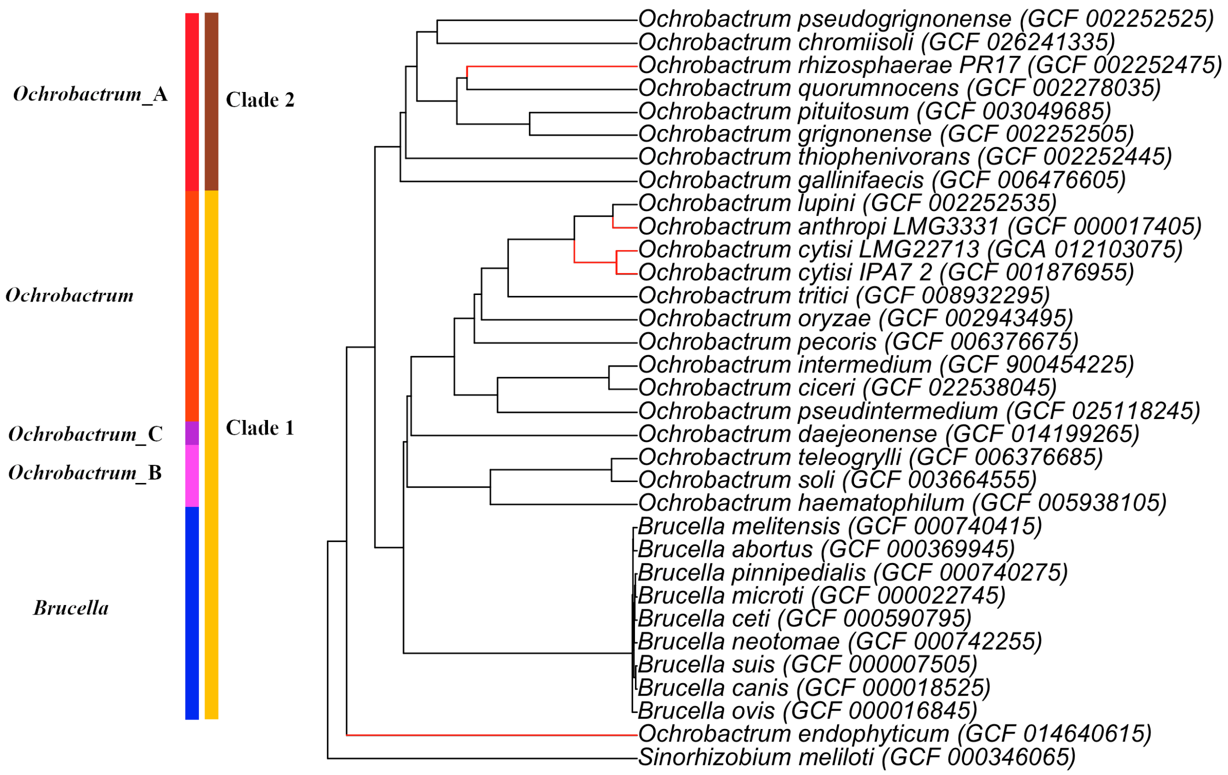

Figure S3. Phylogenetic tree (UPGMA) of bacteria belonging to the genera *Brucella* and *Ochrobactrum* (*Brucella*/*Ochrobactrum* group) based on average nucleotide identity (ANI).

The division of species into Clade 1 and Clade 2 is given in accordance with Leclercq et al. (2020); the division into groups *Brucella*, *Ochrobactrum*, *Ochrobactrum*_A, *Ochrobactrum*_B, and *Ochrobactrum*_C is given in accordance with GTDB-Tk data (https://github.com/kbaseapps/kb_gtdbtk).

The red branches on the phylogenetic tree show strains of the genus *Ochrobactrum* for which the O-polysaccharide structures have been previously described.

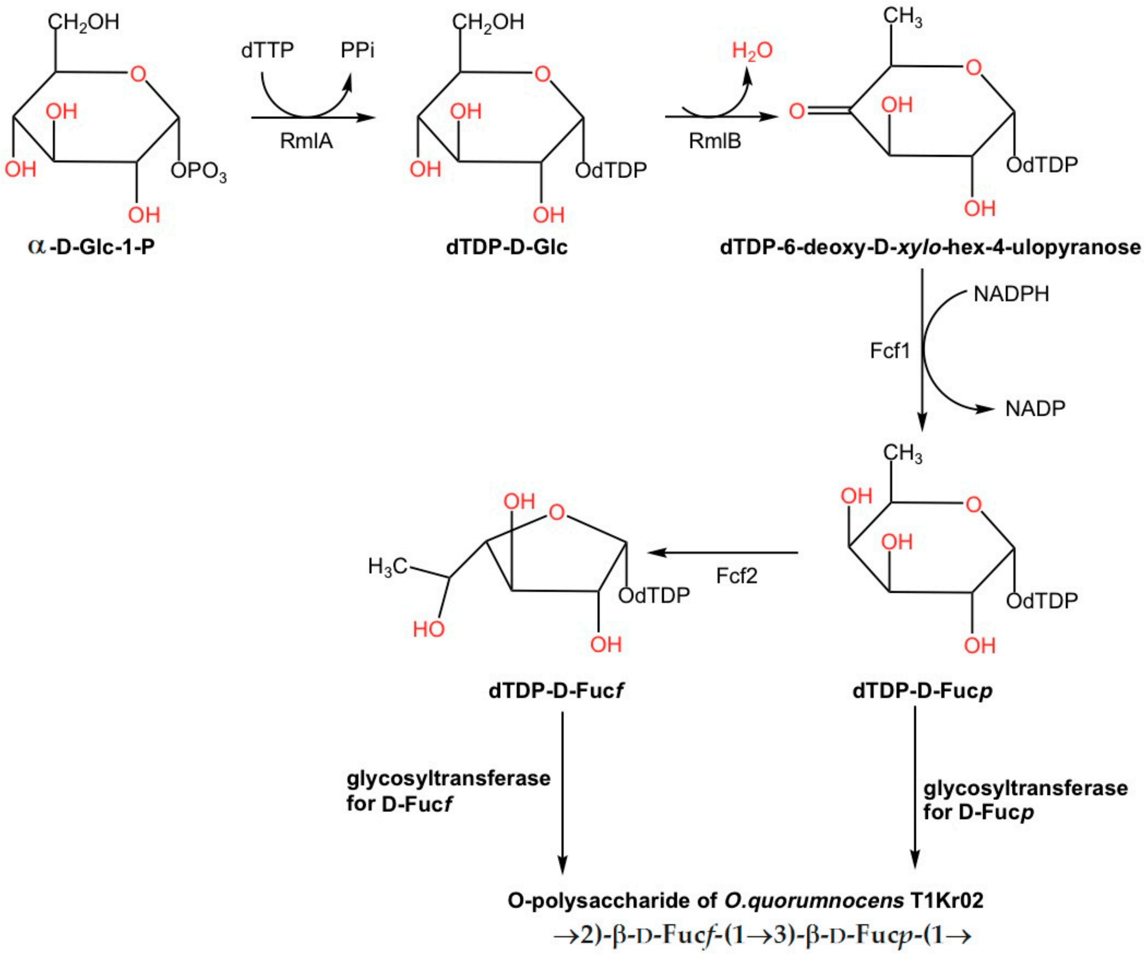

Figure S4. The putative biosynthesis pathway of nucleotide-activated residues of D-fucopyranose and D-fucofuranose for the O-polysaccharide of *Ochrobactrum quorumnocens* T1Kr02.

RmlA (EC 2.7.7.24) – glucose-1-phosphate thymidylyltransferase;

RmlB (EC 4.2.1.46) – dTDP-glucose 4,6-dehydratase;

Fcf1 (EC 1.1.1.266) – dTDP-4-dehydro-6-deoxyglucose reductase;

Fcf2 (EC 5.4.99.59) – dTDP-fucopyranose mutase.

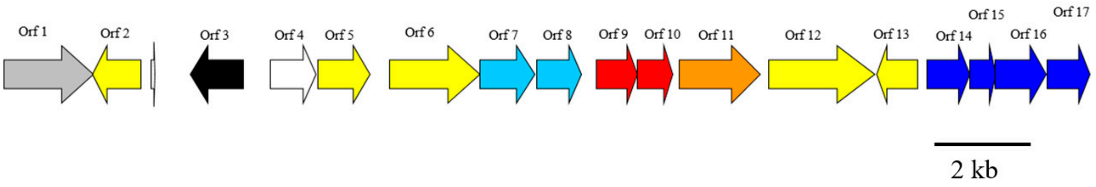

Figure S5. O-antigen biosynthesis gene cluster in the genome of the *Ochrobactrum quorumnocens* strain RPTAtOch1 (acc. number VYXQ01000001), phylogenetically close to the *Ochrobactrum quorumnocens* strain T1Kr02.

Red arrows: genes of the O-polysaccharide processing *wzm* (Orf9) and *wzt* (Orf10); yellow arrows: glycosyltransferase genes; blue arrows: genes for the biosynthesis of dTDP-L-rhamnose – *rml*A (Orf14), *rml*B (Orf16), *rml*C (Orf15), and *rml*D (Orf17); blue arrows: genes for the biosynthesis of dTDP-D-fucopyranose (*fcf*1, Orf 8) and dTDP-D-fucofuranose (*fcf*2, Orf7); orange arrow: gene of methyltransferase *wbe*T (Orf11); black arrow: gene of acetyltransferase; gray arrow: *wzd* gene involved in initiation of repeat unit biosynthesis.

The biosynthesis of dTDP-D-fucopyranose (Fig. S4) requires the functioning of the enzymes RmlA (Orf14), RmlB (Orf16), and Fcf1 (Orf8). The biosynthesis of dTDP-D-fucopyranose additionally requires the enzyme Fcf2 (Orf7).

The glycosyltransferase (Orf6) and *fcf*2 (Orf7) genes have an overlap of 1 bp, which indicates the functional connectivity of the products of these genes. Presumably, Orf6 is a glycosyltransferase that adds D-fucopyranose residues to the O-polysaccharide repeat unit.
